# Cell Therapy in the Treatment of Coronary Heart Disease

**DOI:** 10.3390/ijms242316844

**Published:** 2023-11-28

**Authors:** Elena V. Chepeleva

**Affiliations:** 1 Federal State Budgetary Institution National Medical Research Center Named after Academician E.N. Meshalkin of the Ministry of Health of the Russian Federation, 15, Rechkunovskaya Str., 630055 Novosibirsk, Russia; e_chepeleva@meshalkin.ru; 2 Research Institute of Clinical and Experimental Lymphology—Branch of the Institute of Cytology and Genetics Siberian Branch of Russian Academy of Sciences, 2, Timakova Str., 630060 Novosibirsk, Russia

**Keywords:** coronary heart disease, ischemia, regenerative medicine, stem cells, induced pluripotent stem cells, cell therapy

## Abstract

Heart failure is a leading cause of death in patients who have suffered a myocardial infarction. Despite the timely use of modern reperfusion therapies such as thrombolysis, surgical revascularization and balloon angioplasty, they are sometimes unable to prevent the development of significant areas of myocardial damage and subsequent heart failure. Research efforts have focused on developing strategies to improve the functional status of myocardial injury areas. Consequently, the restoration of cardiac function using cell therapy is an exciting prospect. This review describes the characteristics of various cell types relevant to cellular cardiomyoplasty and presents findings from experimental and clinical studies investigating cell therapy for coronary heart disease. Cell delivery methods, optimal dosage and potential treatment mechanisms are discussed.

## 1. Introduction

Coronary heart disease (CHD), also known as ischemic heart disease, is a common condition that is the leading cause of mortality and disability in adults [1,2]. Myocardial ischemia occurs when the oxygen demand of the myocardial tissue exceeds the ability of the coronary arteries to deliver it due to a primary reduction in coronary blood flow and/or a reduction in coronary reserve. The term “myocardial infarction” (MI) is used when there is evidence of myocardial necrosis due to prolonged acute ischemia [3]. While the development of surgical and pharmacological methods for treating CHD has increased patient survival rates, individuals who have suffered from an MI remain at a high risk of experiencing post-infarction complications and a decline in their quality of life [4]. After an MI, the heart activates local compensatory mechanisms that result in decreased myocardial metabolism, which can lead to the development of heart failure and potentially, sudden death [5]. Patients with severe left ventricular myocardial dysfunction, with an ejection fraction of less than 35% due to post-infarction cardiac remodeling, represent the most severe category of patients among those with CHD [6,7].

**Figure 1:**
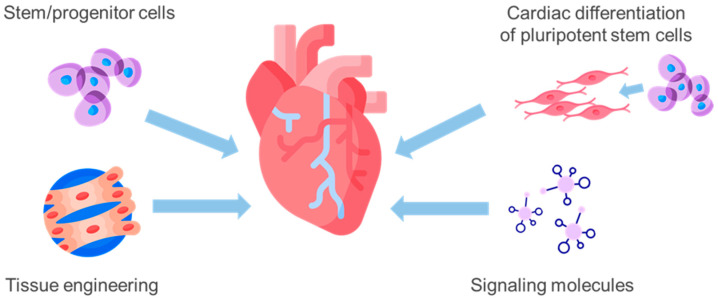
Basic approaches to cell therapy for coronary heart disease: transplantation of adult stem/progenitor cells releases cardioprotective factors through paracrine effects; cardiomyocytes or their precursors derived from induced pluripotent stem cells can also be transplanted to replace lost myocytes; tissue engineering approaches combining cells with biomaterials can create functional heart tissue in vitro for transplantation; small molecules such as growth factors or microRNAs can be delivered to promote cardiomyocyte proliferation or angiogenesis for wound healing.

The primary treatments for CHD include pharmacotherapy and surgical revascularization of the myocardium [8]. Although timely reperfusion and thrombolytic therapy can slow the adverse progression of cardiac remodeling, these treatments cannot completely restore myocardial structure, and the ultimate goal of treatment is to achieve remission [9]. When the disease reaches advanced stages, heart transplantation remains the only effective treatment method. However, the possibilities of implementing heart transplantation are restricted by strict selection criteria and limited availability of donor organs. As a result, the field of cardiac surgery has focused on finding and implementing new approaches to restore blood circulation in ischemic areas of the myocardium for many years [10]. Cell therapy methods show great potential for restoring myocardial function, providing patients with an additional restorative treatment option in conjunction with currently used surgical and pharmacological methods (Figure 1).

The first trials of cell therapy for CHD were conducted nearly two decades ago, when the primary method was to transplant various cell populations derived from adult tissues, such as skeletal muscle myoblasts [11,12], bone marrow mesenchymal stromal cells (MSCs) [13,14], hematopoietic stem cells (HSCs) [15,16] and endothelial progenitor cells (EPCs) [17]. In the early 2000s, a group of scientists led by P. Anversa described c-Kit-positive cardiac cells and their potential for treating CHD [18]. While it was initially thought that these cells could differentiate into cardiomyocytes, endothelial cells and smooth muscle cells, this hypothesis has yet to be confirmed [19,20]. Currently, the regenerative effect of c-Kit-positive cells during transplantation is not fully understood and is thought to be due to the secretion of paracrine factors and stimulation of therapeutic angiogenesis [20]. The development of protocols for transplantation of cardiomyocytes or their precursors derived from in vitro differentiation of induced pluripotent stem cells (iPSCs) to replace lost myocytes is another approach to restore cardiac tissue during ischemic injury [7,21]. Transplantation methods are being developed using various matrices or tissue-engineered structures to enhance cell survival in the recipient.

To date, there are over 100 clinical trials of cell therapy for acute MI and over 90 for chronic ischemic cardiomyopathies [21,22]. The majority of these studies have shown that this type of therapy is safe, regardless of the specific cell product studied, delivery route, dosing protocol, or patient characteristics. However, the optimal cell type for transplantation in cell therapy for CHD has yet to be determined, and the characteristics of these cells and the mechanisms by which they activate reparative processes in the myocardium are poorly characterized, despite the wealth of research in this area. This article reviews research on the use of various cell types in the treatment of CHD to examine the challenges of experimental and clinical trials and to assess the potential for advancing cell therapy for myocardial repair.

A search for relevant data was conducted using databases such as PubMed and Scopus. The following keywords were utilized: heart disease, ischemia, regenerative medicine, stem cells, pluripotent stem cells, cell therapy, and myocardial repair. Experimental and review articles were included in the search.

## 2. Non-Cardiac Origin Cells for Cardiac Cell Therapy

### 2.1. Skeletal Myoblasts

Skeletal muscle myoblasts have been the first cell type to be investigated in preclinical and clinical studies of cell therapy for CHD [23]. These cells possess the advantage of being easily obtainable from the patient, as well as having the ability to differentiate into contractile cells [24]. Myoblasts are derived from myosatellite cells present beneath the basal lamina of skeletal muscle fibers [12]. They are identified by the expression of Pax7, Pax3, c-Met, M-cadherin, CD34, syndecan-3, and calcitonin [25]. In mature muscle, satellite cells are typically quiescent [26]. However, injury activates satellite cells and induces MyoD expression, leading to cell cycle entry and the generation of myogenic precursors or myoblasts [27]. After multiple cell divisions, myoblasts exit the cell cycle, fuse with each other, and create multinucleated myotubes that develop into mature muscle fibers. The ability of myoblasts to form muscle fibers in regenerating muscle and ectopic muscle fibers in non-muscle tissues is a potential therapeutic approach for Duchenne muscular dystrophy, urological dysfunction, and heart failure [28].

Early uncontrolled clinical studies have reported that transplanted skeletal myoblasts engraft into the heart and result in better cardiac performance in patients [11]. However, a subsequent randomized controlled trial of transepicardial autologous skeletal myoblast transplantation during coronary artery bypass grafting in 97 patients with severe left ventricular dysfunction did not reproduce these findings. At 6 months post-surgery, patients who received cell treatment did not demonstrate any significant differences in the functional state of the left ventricle compared to the control group. However, there was an increased occurrence of ventricular tachyarrhythmia, which resulted in early termination of the study [12]. Similar results were observed in the SEISMIC clinical trial, where transendocardial skeletal myoblast transplantation was used. Four years following the procedure, no discrepancies were observed in the left ventricular function between the experimental and control groups [29]. It is generally accepted that the transplantation of skeletal myoblasts is not sufficient to provide a complete electrical conduction system in the heart [30].

### 2.2. Hematopoietic Stem Cells

One common type of stem cell utilized in cell therapy is HSCs, which comprise no more than 0.1% of unfractionated mononuclear cells found in bone marrow [31]. HSCs have the ability to self-renew and generate multipotent progenitors that differentiate in a sequential fashion to produce various specialized cells, such as lymphocytes, dendritic and natural killer cells, megakaryocytes, erythrocytes, granulocytes, and macrophages [32]. During the process of differentiation, HSCs demonstrate distinct antigenic characteristics that are associated with their properties and functions. These antigens aid in the identification of stem cell subpopulations and enhance the outcomes of HSCs transplantation by increasing the purity of the cell product utilized in allografts [33]. The surface antigen CD34 is expressed by human HSCs and is commonly used as a marker in clinical settings to identify and quantify the population of progenitor cells to be infused [34]. Additional surface markers such as CD90, CD38, c-Kit, CD105, and HLA-DR are used for evaluation of cell maturity [33]. The combined use of these markers provides valuable information for the isolation and purification of HSCs subpopulations. However, phenotypic characterization of HSCs provides only a partial picture of their functional activity (especially after ex vivo expansion), so that the best subset of HSCs for the treatment of CHD remains to be identified. [31,33].

In 2001, a study evaluated the effect of intramyocardial administration of HSCs during modeling of MI in mice [35]. The researchers observed differentiation of the transplanted cells into cardiomyocytes and endothelial cells, resulting in a relative enhancement of cardiac performance in the animals. Subsequent studies on the use of HSCs for cell therapy in CHD failed to show a beneficial effect of cell transplantation and demonstrated the inability of HSCs to differentiate into cardiomyocytes [36,37]. The different results of these studies have been attributed to the use of different cell isolation and culture protocols.

Several clinical studies on the use of HSCs in the treatment of patients with CHD (mainly ischemic cardiomyopathy) have shown that HSC transplantation is completely safe and does not cause any complications, but no significant improvement in cardiac function was observed [15,16,38]. Currently, it is speculated that HSCs may exert a paracrine effect on cardiac tissue by releasing growth factors that promote angiogenesis [21].

### 2.3. Endothelial Progenitor Cells

In 1997, researchers described a group of stem cells from bone marrow known as EPCs [39]. These cells represent a provasculogenic subpopulation of HSCs and display surface markers CD34 and CD133 [23]. EPCs hold promise for the treatment of CAD due to their potential to directly contribute to blood vessel formation in recipient tissues and to stimulate angiogenesis through paracrine signaling. Systemic administration of EPCs has been found to increase neovascularization in ischemic tissues [40,41].

The clinical trial RENEW was dedicated to intramyocardial transplantation of autologous EPCs to patients with refractory angina. The authors confirmed the safety of using these cells and observed improvements in some functional parameters of the left ventricle [17]. The PERFECT clinical trial reported a reduction in scar size following MI and an improvement in segmental myocardial perfusion due to intramyocardial transplantation of autologous EPCs during coronary artery bypass grafting in patients with acute MI. However, the study did not uncover any variation in left ventricular ejection fraction after cell transplantation [42].

### 2.4. Mesenchymal Stem Cells 

Another group of bone marrow stem cells consists of MSCs or fibroblast colony-forming units. MSCs are characterized by their ability to adhere to scaffolds and to differentiate into osteoblasts, adipocytes, and chondrocytes under specific in vitro conditions, and they express specific cellular markers such as CD73, CD90, and CD105, but lack CD14, CD45, CD34, and HLA-DR [23]. It should be noted that MSCs have a low level of expression for the major histocompatibility complex (MHC II) molecule, making them suitable for allogeneic transplantation [43]. It is believed that MSCs are capable of suppressing the production of inflammatory cytokines and have cytoprotective and angiogenic effects [44,45]. As a result, MSCs offer a promising source of cell cultures for treating CHD.

Studies of intramyocardial MSCs transplantation in laboratory animals with experimentally induced MI have shown reduced area of cardiosclerosis and increased peri-infarction angiogenesis [46,47,48]. A recent meta-analysis, comprising 58 preclinical studies, indicated that transplantation of MSCs in animal models of acute MI and chronic CHD resulted in a decrease of around 7% in post-infarction scar size and an approximately 11% enhancement in cardiac contractile function [49].

Intravenous administration of MSCs to patients after MI was investigated in a clinical trial in 2009 [13]. The study showed that patients who underwent treatment had a reduced risk of developing arrhythmias, and there was a relative improvement in left ventricular functional characteristics 3 months after the injections. The POSEIDON clinical trial, which investigated the use of MSCs in patients who had undergone coronary artery bypass grafting for ischemic cardiomyopathy, yielded comparable results [14]. In the C-CURE clinical trial, researchers treated MSCs with a cytokine cocktail intended to induce cardiogenic differentiation, resulting in the development of cardiopoietic stem cells. Patients with ischemic cardiomyopathy who received transendocardial injection of these cells in the left ventricle displayed a relative increase in cardiac contractile function as compared to the control group [50]. The PROMETHEUS clinical trial demonstrated a reduction in the area of cardiosclerosis and improved cardiac contractile function in patients who underwent intramyocardial transplantation of autologous MSCs [51]. Similar results were found in other clinical studies that used transendocardial injections of MSCs, which also led to a relative reduction in the area of cardiosclerosis in the heart [52,53,54]. In certain clinical studies, no discernible distinctions were discovered between patients receiving MSCs and those in the control group [55,56,57].

While bone marrow is the primary source of MSCs, they can also be extracted from almost any organ [58]. Particular emphasis has been placed on cells derived from adipose tissue, and animal studies have demonstrated an increase in cardiac contractile function and the promotion of angiogenesis following the administration of MSCs derived from adipose tissue [30,59].

## 3. Cardiac Origin Cells for Cardiac Cell Therapy

### 3.1. Cardiac c-Kit+ Cells

In 2003, P. Anversa and colleagues proposed the presence of regional cardiac stem cells expressing the c-Kit (CD117) stem cell factor receptor in the postnatal myocardium. They based this proposal on c-Kit being a marker for HSCs, which implies a population of progenitor cells exists in the heart [18]. In their study, the researchers demonstrated the lack of expression of HSCs markers (CD34, CD45) in c-Kit+ cardiac cells and their ability to differentiate in vitro towards cardiomyocyte, endothelial, and smooth muscle cell directions [18]. The same group of investigators showed that transplantation of c-Kit+ cells into the ischemic hearts of rats resulted in the recovery of up to 70% of the affected myocardium, probably due to the in vivo generation of new cardiomyocytes from donor cells [18]. Subsequently, similar results were observed in experiments in dogs and minipigs [60,61].

The discovery of regional cardiac stem cells initially generated excitement in the scientific community, but subsequent research by independent groups failed to demonstrate the differentiation potential of c-Kit+ cells toward cardiomyocytes, both in vitro and in vivo after transplantation into the heart [62,63,64]. Most experimental studies have observed a relative restoration of cardiac contractile function after transplanting c-Kit+ cells into ischemic myocardium. An analysis of 80 preclinical studies evaluating the efficacy of autologous and allogeneic c-Kit+ cell transplantation in acute MI showed a mean increase in left ventricular ejection fraction of almost 11% compared to control groups, with a greater effect observed in small animals than in large animals [65].

The CONCERT-HF clinical trial demonstrated improvements in functional and structural characteristics of the left ventricle in patients with heart failure due to ischemic cardiomyopathy following co-transplantation of autologous MSCs and c-Kit+ cardiac cells [66].

Based on Roberto Bolli’s 2015 hypothesis, c-Kit+ cells found in the adult heart originate from the epicardium and are considered regional mesenchymal cells based on their immunophenotype, which includes expression of the mesenchymal markers CD90 and CD105 [67]. The beneficial effect of c-Kit+ cell transplantation on myocardial tissue recovery after ischemic injury, as demonstrated in numerous studies, may be due to the release of cytokines (TCA-3, SDF-1), vascular growth factors (VEGF, HGF, erythropoietin, FGFb, osteopontin, SCF), regulatory factors of cardiac differentiation (activin A, Dkk homolog-1, TGF-β), and the promotion of angiogenesis at the injection site [20,23,68,69].

### 3.2. Cardiosphere-Derived Cells 

The term “cardiosphere” was introduced in 2004 to describe undifferentiated cells obtained with enzymatic treatment of atrium wall explants [70]. Cardiospheres are a heterogeneous cell population comprised of a core of c-Kit+ cells, several layers of cells expressing connexin 43 (the primary gap junction protein in the heart), and an outer layer consisting of CD105-expressing cells on the surface [71,72].

Despite significant and increasing interest in this particular cell type, there remains limited understanding of their origin. It is believed that cardiospheres arise from stromal cells present in the myocardium and proliferate during primary culture to form cardiospheres in vitro [73].

Research on the use of cardiosphere-derived cells (CDCs) for the treatment of experimental MI in rodents has demonstrated a relative improvement in the functional characteristics of the heart [70]. Studies comparing the efficacy of treating experimental MI in mice using bone marrow MSCs, adipose tissue MSCs, HSCs, and CDCs revealed that the group that underwent CDCs transplantation demonstrated the most significant recovery of cardiac muscle [74]. The authors attributed this advantage to the optimal conditions for transplanted cell functioning in the myocardium created by intercellular interactions within the cardiosphere. Meanwhile, intramyocardial transplantation of CDCs failed to demonstrate a therapeutic effect in several animal studies with artificially induced MI [75,76].

In 2007, a clinical protocol was established for extracting human cardiosphere cells through endomyocardial biopsy [71]. Experiments modeling ischemia-reperfusion in pigs have demonstrated the therapeutic potential of human CDCs [77]. The first phase of the CADUCEUS clinical trial investigated the intracoronary administration of autologous CDCs to post-MI patients [78]. The authors obtained myocardial slices from endocardial biopsy and subsequently isolated cardiospheres. The cardiospheres were administered to patients 2 to 4 weeks after MI, and the intervention was found to be safe for use in humans. The magnetic resonance imaging results after 3 months indicated that patients who underwent treatment experienced a decrease in the area of cardiosclerosis and an increase in healthy myocardial mass and left ventricular wall thickness. However, there were no significant differences in ejection fraction observed [78]. Subsequent clinical trials in the United States employed allogeneic CDCs, revealing comparable therapeutic results, and greater potential for use as a cell product for treating CHD [73]. These trials encompassed ALLSTAR (mild heart failure and low ejection fraction in post-MI patients) [79], DYNAMIC (severe heart failure and low ejection fraction in post-MI patients) [80], HOPE-Duchenne, and HOPE-2 (patients with Duchenne muscular dystrophy and cardiomyopathy) [81,82]. The completed studies confirmed the safety of using CDCs in clinical settings and demonstrated their potential for disease-modifying biological activity [73].

The CDCs’ efficiency is mainly based on the “paracrine hypothesis”. This suggests that the transplanted cells produce vesicles that contain biologically active molecules [73,83], leading to therapeutic effects such as antifibrotic [84], antiapoptotic [85,86], angiogenesis stimulation [87,88], modulation of inflammatory processes, and oxidative stress control [88,89], as well as cardiomyocytes re-entering the cell cycle stimulation [90].

### 3.3. Epicardium-Derived Progenitor Cells

The epicardium, consisting of epicardial mesothelial cells, collagen, and elastic fibers, is the outer layer of the heart wall in close proximity to the myocardium. The formation of a functional heart during embryogenesis is a result of interactions between the cardiogenic mesoderm, cardiac neural crest, and proepicardium [91]. In nearly all vertebrates, the proepicardium is a cluster of progenitor cells located at the venous pole of the embryonic heart [92]. During embryogenesis, epicardial progenitor cells (EPDCs) emerge in the epithelial–mesenchymal transition and contribute to the growth of cardiac fibroblasts, smooth muscle cells, and pericytes [93]. From the latter half of embryonic development, epicardial mesothelial cells progressively lose their capability to proliferate, convert into a layer of dormant epicardial cells, and persist in the postnatal period [94].

It has been widely believed that EPDCs are functionally inactive in the adult heart. However, recent studies suggest that they may contribute to the regeneration of cardiac tissue during age-related changes or after myocardial damage [95]. A coherent and firmly established comprehensive understanding of these factors is yet to be established. However, diverse animal models have demonstrated that the epicardium participates in bidirectional paracrine signaling, stimulating the proliferation of cardiomyocytes, attracting macrophages, and promoting the development and maturation of coronary vessels [96].

Current evidence suggests that the epicardium is composed of distinct subpopulations of cells with different abilities to upregulate embryonic gene expression (*Wt1*, *Raldh2*, *Tbx18*), promote proliferation, and induce epithelial–mesenchymal transition in response to cardiac injury, which has important clinical implications and potential therapeutic applications [97]. Once activated, the epicardium releases mitogenic signaling factors to cardiomyocytes, promoting cardiac regeneration [98,99]. TGFb, PDGF, FGF, IGF, BMP, retinoic acid, Notch, NF-κB, Hippo/Yap, Sonic Hedgehog, Wnt/β-catenin signaling pathways, and extracellular matrix proteins are the major regulators of this process that have been identified to date [97]. It has been shown that epicardial mesothelial cells in the adult human heart are able to give rise to cardiac stromal cells that express the c-Kit receptor during the epithelial–mesenchymal transition [100]. Thus, EPDCs’ regenerative potential is regulated by paracrine factor release and specific reactions to activated signaling pathways. However, direct participation in cardiomyocyte formation from EPDCs was not observed [93]. To initiate regenerative processes in the damaged adult heart, activation of the epicardium is required, with a focus on neovascularization and regeneration, while inhibiting the fibrocytic pathway and pro-inflammatory elements.

The first pharmacological attempt to influence these processes involved the administration of thymosin β4, which plays a role in the vascularization of the neonatal heart [101]. In experiments that modeled MI in mice, results indicated that pre-treatment with thymosin β4 activated quiescent EPDCs, which subsequently raised the angiogenesis levels in the targeted tissues [101,102]. However, activation of EPDCs was not observed when administering thymosin β4 during the early post-infarction period [103]. Other researchers have utilized prokineticin-1 and VEGF-A for the same purposes [104,105]. Additionally, a study has revealed that subepicardial implantation of collagen patches containing Fstl-1, a protein secreted by epicardial cells responsible for myocardial regeneration, partially improves cardiac contractile function in mice and pigs with induced MI [106].

Thus, the effects on the epicardium represent a novel approach to addressing the intricate problem of therapeutically regenerating CHD. One direction involves developing tissue-engineered patches that incorporate the necessary extracellular matrix components and are filled with EPDCs. Another option is to trigger repair processes by utilizing viral vectors that target mature and progenitor epicardial cells.

## 4. Pluripotent Stem Cells for Cardiac Cell Therapy

### 4.1. Embryonic Stem Cells

Embryonic stem cells (ESCs) come from the inner cell mass of the blastocyst during early embryonic development. ESCs are pluripotent, which means they can differentiate into all three primary germ layers: ectoderm, endoderm, and mesoderm [107]. Under carefully selected culture conditions, ESCs can differentiate into various types of adult somatic cells, making them an exciting therapeutic option for the treatment of coronary artery disease. However, several limitations decrease the feasibility of ESCs in cell therapy. Firstly, undifferentiated pluripotent stem cells possess oncogenic potential and may result in the formation of tumors in recipients upon transplantation. Secondly, ESCs are allogeneic cells that the body may reject following transplantation. Finally, ESCs’ use is restricted by the legislation of many countries due to various moral and ethical issues [108,109].

During the initial stages of researching the potential use of ESCs transplantation in treating CHD, there was a belief that they could differentiate into cardiomyocytes within the cardiac tissue microenvironment [110]. However, this assumption was later disproven when undifferentiated ESCs were intramyocardially injected, leading to the formation of teratomas [111]. In later stages, researchers directed their focus to differentiating ESCs toward cardiomyocyte generation and transplanting the resulting cells into the myocardium [112]. Multiple experimental studies demonstrated the participation of cardiac derivatives from human and mouse ESCs in the restoration of cardiac tissue in experimental animals [113,114,115,116]. The ESCORT clinical trial demonstrated the short-term safety of transplantation of human cardiac ESC-derived cells in patients with severe left ventricular dysfunction after myocardial infarction. No tumors or arrhythmias were detected during the follow-up period, and three out of six patients developed clinically asymptomatic alloimmunization [117].

### 4.2. Induced Pluripotent Stem Cells

**Figure 2:**
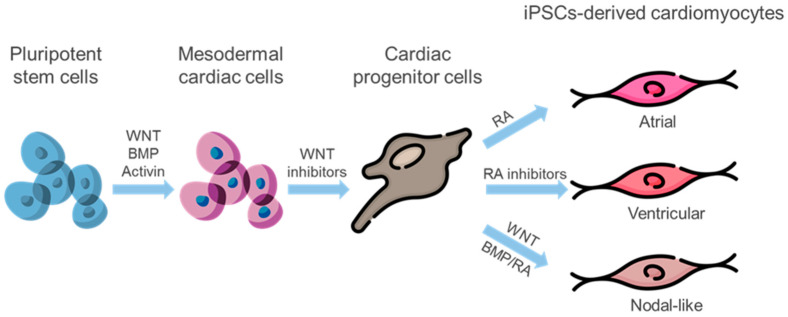
Generating subtype-specific cardiomyocytes: Pluripotent stem cells are induced to cardiac mesoderm through activation of Wnt and BMP signaling. Administration of Wnt inhibitors leads to the commitment of cardiac progenitors. RA signaling guides cardiac progenitors towards atrial subtype commitment, while inhibition of RA signaling promotes ventricular lineage differentiation. Furthermore, RA and BMP act together to induce cardiac progenitor cells to become nodal-like pacemaker cells. Wnt signaling additionally promotes the specification of pacemaker cells from cardiac mesoderm cells. BMP—bone morphogenetic protein, RA—retinoic acid.

In 2006, K. Takahashi and S. Yamanaka demonstrated the ability to reprogram differentiated somatic cells into induced pluripotent stem cells (iPSCs) [118]. This groundbreaking discovery provides a solution to the problems that arise when using ESCs. First, there is no need for the use of human embryos, and second, there are no problems with rejection and histocompatibility. Cells formed after differentiating modified iPSCs can be used for patient-specific therapy. Directed differentiation of induced pluripotent stem cells (iPSCs) into cardiomyocytes may be achieved through BMP, TGFb/activin/NODAL, Wnt, and FGF signaling pathways [119]. Although iPSCs-derived cardiomyocytes have an immature and fetal-like phenotype, they exhibit a combination of atrial, ventricular, and nodal-like electrophysiological properties. Differentiation protocols utilizing a blend of chemicals and growth factors have resulted in the creation of subtype-specific cardiomyocytes [120] (Figure 2).

The resulting cells can be used to restore the depleted contractile components of the myocardium [121,122]. Upon differentiation, cardiomyocytes exhibit contractility and excitability in response to sympathetic and parasympathetic nervous system signals; however, there is currently no optimal approach for the production and transplantation of fully functional cardiac tissue capable of generating synchronized contractions and rhythmic activity from in vitro cultured cells [123].

Some studies have demonstrated improvements in cardiac function and a reduction in fibrosis formation following implantation of iPSCs-derived cardiovascular progenitor cells or cardiomyocytes in acute and subacute stages of MI in rodent models [124,125,126,127]. The effectiveness of these cells in improving cardiac function, revascularization, and/or remuscularization of infarcted hearts is further confirmed in non-human primates [128,129,130] and pigs [131,132]. The safety of early clinical trials using human iPSCs-derived cardiomyocytes in patients with severe ischemic cardiomyopathy has been reported [117,133].

## 5. Mechanisms of Action for Cardiac Cell Therapy

The study of the mechanisms by which different types of transplanted cells regulate the regeneration of ischemic heart tissue is crucial for the advancement and improvement of cell therapy methods, and it is essential that these mechanisms be explored for a deeper understanding of the potential utility of cell therapy in the treatment of CHD. Both direct integration into the myocardium to compensate for cardiomyocyte or endothelial cell loss and indirect paracrine mechanisms may be involved in the ability to repair damaged tissue [23,134]. Direct cell or tissue replacement provides the most straightforward approach to remuscularize the myocardium and restore function after acute infarction. For irreversible fibrosis or remodeling associated with chronic injury, a much larger and more complex indication than acute or subacute MI, remuscularization may be the only effective approach. However, at this stage of development, replacement therapies are still elusive and exploratory [21]. As evidenced by numerous in vitro and in vivo studies, paracrine signaling is the fundamental mechanism mediating the beneficial effects of cell therapy [83,135].

Through paracrine signaling, transplanted cells are able to activate various signaling pathways and influence the surrounding cardiac tissue, independent of the establishment of functional intercellular contacts with the recipient cells [136]. Biologically active molecules including TGFb, VEGF, SDF1, EGF, and HGF can be secreted into the intercellular space or bloodstream by transplanted cells [137,138]. Consequently, the release of cytokines or extracellular vesicles is a systemic event that stimulates various regenerative processes, such as neovascularization, reduced apoptosis of endogenous cardiomyocytes, activation of tissue progenitor cells, or recruitment of cells responsible for repair of damaged tissues [136,139]. The secretome characteristics of transplanted cells and their ability to regenerate myocardium are strongly influenced by the health status and age of the donor, as well as the methods used to obtain and prepare the cellular material prior to transplantation [23].

Neovascularization plays a critical role in regenerative processes by providing ischemically damaged tissue with the necessary nutrients and oxygen for replenishment, a process that is essential for tissue recovery. In a study using a mouse model of MI, researchers found that human CDCs released pro-angiogenic factors such as VEGF, HGF, and IGF1 after transplantation, and noted a 20% increase in neovascularization in the peri-infarct region following subcutaneous cell injection, which was mainly related to paracrine signaling [87]. A recent study confirmed the stimulatory effect of CDCs on angiogenesis in mice and identified endoglin (CD105) as an important mediator of this paracrine-induced neovascularization [140]. It was also shown that transplantation of rat c-Kit+ cardiac cells into the peri-infarct area significantly enhanced angiogenesis by secreting VEGF [141].

MSCs derived from different sources have the ability to release proangiogenic factors, which aids in the development of new blood vessels [136,142]. In a study by L. Wang et al., adipose tissue-derived MSCs were found to produce VEGF, HGF, and IGF1 in vitro and to increase capillary density in the peri-infarct area after transplantation into rats [143]. Because direct differentiation of injected cells into endothelial cells was very low in this study (<1%), the authors concluded that increased neovascularization was mainly stimulated by paracrine cytokine release [143]. The therapeutic efficacy of the bone marrow MSCs’ secretome was also demonstrated by L. Timmers et al., who intravenously administered conditioned medium of human MSCs to pigs with experimental MI [144]. After three weeks, the animals treated with the conditioned medium had a significantly greater number of capillaries in the border region than the control group [144].

Preclinical studies using HSCs and EPCs have demonstrated significant improvements in capillary density and increased neovascularization in ischemic cardiac tissue after MI [145,146,147]. Research has also shown that HSCs and EPCs can secrete proangiogenic factors such as VEGF, FGFb, IGF1, HGF, and SDF-1α [148,149].

## 6. Cell Dosing and Delivery

The goal of delivering cells to the recipient’s heart is to ensure that a sufficient number of viable donor cells can enter the damaged areas of the myocardium shortly after transplantation and remain for a long time, effectively integrating and functioning [21]. Currently, intramyocardial, intracoronary, and intravenous administration are commonly used interchangeably, as there is no optimal method for cell transplantation into the heart [21,150].

The intracoronary delivery method enables direct delivery of cells to the affected regions of the myocardium via the coronary arteries [151]. This approach is usually employed during primary percutaneous coronary intervention for patients with acute MI. Using retroviral transfection with plasmids expressing GFP, B. Dawn and colleagues have demonstrated the capacity for transcoronary migration of transplanted c-Kit+ cardiac stromal cells and their subsequent engraftment into the myocardium of the recipient animal [152]. However, intracoronary administration results in washout of cells into the systemic circulation, leading to insignificant engraftment in the heart, which may be sufficient for paracrine effects but not for myocardial repair during replacement therapy [150]. Cardiomyocytes obtained through directed differentiation of iPSCs cannot be delivered in large quantities via intracoronary infusion due to the risk of microvascular occlusion [21].

Intravenous administration through peripheral injection represents the least invasive and simplest method, obviating the need for surgical or endoscopic interventions. The efficacy of intravenous administration of stem cells is dependent on the existence of a homing effect, i.e., an increase in the migration of cells to the site of myocardial damage [153]. However, it should be noted that the majority of transplanted cells from the systemic circulation enter the lungs, while only a small quantity reaches the heart, liver, and spleen [154].

Intramyocardial delivery of cells to the heart can be accomplished using two methods: transendocardial and transepicardial. The latter approach is utilized during open-heart surgery and boasts accurate delivery of cells to the intended area of the myocardium through direct visualization of the ischemic area. Transendocardial intramyocardial administration, meanwhile, presents as an efficient and minimally invasive means of cell transplantation. Cells are introduced using a special system (NOGA system), which includes a catheter with a needle and an electromechanical mapping system [155]. Transendocardial and transepicardial methods of transplanting autologous skeletal myoblasts were found to be equally effective in an experimental study modeling myocardial infarction in minipigs [156]. In a study comparing transplantation techniques for CD34+ autologous bone marrow stem cells in patients with non-ischemic dilated cardiomyopathy, intramyocardial injection resulted in higher engraftment rates in the heart than intracoronary injection [157]. However, intramyocardial injection also carries a risk of myocardial perforation [21].

Cells survive relatively poorly with current methods of cell transplant, ranging from 10 to 15 percent regardless of the number of cells implanted [158]. The investigation of the relationship between the survival of transplanted cells and the efficacy of cell therapy requires the precise monitoring of the number, distribution and fate of injected cells, and analysis of the correlation of these variables with changes in cardiac functional parameters [159]. To date, numerous studies have been conducted on experimental animals with ischemic cardiomyopathy, including mice, rats, and pigs [19,159,160,161,162,163,164,165]. These studies evaluated the extent of cell engraftment using histological methods, in vivo imaging, or molecular genetic analysis of heart tissue explants. While these studies generally showed improvement in cardiac function and/or reduction in infarct area, they did not demonstrate a correlation between these parameters and the degree of cell survival at various time points [166]. Tracking or estimating the number of cells remaining after transplantation can be technically challenging during clinical trials, as it requires the utilization of non-invasive imaging techniques like MRI, PET, SPECT, and CT. Injected cells must also be pre-labeled with numerous probes and labels, which need further research on their safety prior to use [167]. Histological or molecular analysis of the recipient’s myocardium can only occur in rare cases, specifically when the patient has passed away or a donated heart has undergone transplantation. Therefore, the current focus is on the correlation between the number of cells introduced and the resulting therapeutic benefit.

However, several preclinical and clinical studies on cellular therapy for CHD have failed to establish a clear correlation between the number of cells transplanted and improved cardiac function [168]. Halkos et al.’s study demonstrated that the administration of allogeneic MSCs in pigs with induced MI through intravenous injection of three doses (1, 3, or 10 million) resulted in a more substantial enhancement in left ventricular functional parameters in groups that received higher cell doses (3 and 10 million), as opposed to the control group [169]. Hamamoto and colleagues utilized transendocardial intramyocardial administration of allogeneic MSCs at four different doses (25, 75, 225, or 450 million cells) in a sheep model of induced MI. The study found that lower doses of 25 and 75 million cells had a more pronounced beneficial effect on the heart’s functional state [170]. Schuleri and colleagues conducted a study in which cells were injected into pigs with MI via transepicardial injection during experimental open-heart surgery [171]. The researchers found that administering 200 million autologous MSCs led to a significant reduction in infarct size compared to a lower dose of 20 million. Hashemi et al. found that transendocardial injection of allogeneic MSCs in doses of either 24 or 240 million led to a decrease in infarct area in pigs with induced MI compared to the group receiving a higher dose of 440 million cells [172]. Quyyumi et al. conducted a clinical trial to evaluate the efficacy of intracoronary administration of autologous CD34+ bone marrow cells at different doses (5, 10, or 15 million) in patients with ST-segment elevation MI [173]. The results indicated that patients who received 10 or 15 million cells experienced a significant improvement in myocardial perfusion compared to the control group. In the POSEIDON clinical trial, patients diagnosed with ischemic cardiomyopathy received transendocardial injections of allogeneic or autologous bone marrow-derived MSCs at different doses (20, 100 or 200 million). Regarding improvement of ejection fraction and left ventricular end-systolic volume, an inverse dose-dependent response to cell therapy was observed in this study [14].

It is challenging to compare the outcomes of existing clinical and experimental research studies due to variations in their design, the number of cells provided, the delivery method employed, and the timing of administration. In their meta-analysis, Wang and colleagues reviewed eight randomized controlled trials of MSCs transplantation in patients with MI and subsequent percutaneous coronary artery bypass grafting. Their findings suggest that administration of no more than 10 million cells within one week of MI may optimize restoration of left ventricular systolic function [174]. However, additional clinical trials with carefully designed designs and consideration of individual patient characteristics are essential for a more precise evaluation of the appropriate cell dose and optimal timing of transplantation.

To date, most clinical trials of cell therapy for the treatment of CHD have used a single dose of cells. Transplanted cells are known to engraft with limited success; thus, the re-introduction of cells has been proposed as a means to enhance the recovery of cardiac function [175]. Several clinical studies have shown that repeated transplantation of peripheral blood or bone marrow CD34+ mononuclear cells at intervals of 3 months to 4.6 ± 2.5 years after the initial dose results in improved left ventricular function in patients with ischemic cardiomyopathy compared to a single cell injection [38,176,177,178,179]. However, current invasive delivery methods, such as intracoronary or transendocardial injection, pose challenges in the study of multiple cell doses and in the implementation of placebo controls, leading to significant limitations in the design of clinical trials [175].

## 7. Conclusions and Prospects

Cell therapy, when used in addition to surgical and drug therapies, may help repair damaged heart muscle and improve the quality of life for patients with CHD. However, studies on cell transplantation have generally demonstrated modest, negative, or inconsistent results. Several factors may contribute to this phenomenon, including the methods used to obtain and prepare the cellular material, the method of delivering cells to the body, as well as the study design employed and the diversity of inclusion criteria. In addition, many studies did not systematically adjust for patient characteristics such as older age, comorbidities, and clinical presentation of CHD. The inconsistent results of clinical trials investigating cell therapy for damaged myocardium have highlighted the need for personalized medicine. This approach is confronted with the task of constructing a predictive system to identify patients who will have a favorable response to cell therapy. It is clear that enrollment of patients on the basis of nosological entity alone, taking into account only the stage and/or activity of the disease, is inadequate; the response to cell transplantation is individualistic, and therefore requires the study of patient characteristics.

To date, it is uncertain which cell type is the optimal choice for clinical use. Various cell types have been used in cell therapy for CHD, but there is a lack of preclinical and experimental studies to determine their mechanisms of action after transplantation into the heart. Combination therapies may offer additional benefits due to the different activities of different cell types, but there is currently insufficient data to determine the most effective combinations. The development of protocols for the combined transplantation of cardiomyocytes to replace lost myocardium and paracrine cells to stimulate therapeutic angiogenesis in damaged ischemic tissues is showing promising results in cell therapy for CHD.

The optimal dosage of cellular material for myocardial transplantation remains unknown due to the unexplored issue of selecting the number of transplanted cells for both replacement therapy and therapy using the paracrine effect. An empirical determination of the optimal dosage will be essential until well-conducted and adequately controlled preclinical studies have been carried out. In order to assess the scalability of the number of cells transplanted, it is recommended that preliminary studies be conducted in small animals, followed by studies in larger animals that are physiologically similar to humans.

Despite unresolved questions, the current use of cells for treatment of CHD provides an opportunity for development of new protocols for the collection and preparation of cellular material for transplantation.

## Data Availability

Not applicable.
